# Impact of tobacco and alcohol consumption on disease progression and MRI in people with multiple sclerosis: results of the prospective cohort study NationMS

**DOI:** 10.1177/17562864261464304

**Published:** 2026-07-29

**Authors:** Alex Maximilian Keller, Annika Möhl, Anke Salmen, Ralf Gold, Pia Renk, Luisa Klotz, Stefan Bittner, Vinzenz Fleischer, Friedemann Paul, Klemens Ruprecht, Katrin Giglhuber, Hayrettin Tumani, Gisela Antony, Uwe Zettl, Sven G. Meuth, Antonios Bayas, Corinna Trebst, Brigitte Wildemann, Florian Then Bergh, Daniel Kotz, Christoph Heesen

**Affiliations:** Institute of Neuroimmunology and Multiple Sclerosis, University Medical Center Hamburg-Eppendorf, Martinistraße 52, Hamburg DE-20246, Germany; University Medical Center Hamburg-Eppendorf, Institute of Medical Biometry and Epidemiology, Hamburg, Germany; Department of Neurology, Ruhr-Universität Bochum, Bochum, Germany; Department of Neurology, Ruhr-Universität Bochum, Bochum, Germany; Department of Neurology, Ruhr-Universität Bochum, Bochum, Germany; Department of Neurology with Institute of Translational Neurology, Medical Faculty, University Hospital Münster, Münster, Germany; Department of Neurology, Focus Program Translational Neuroscience (FTN) and Immunotherapy (FZI), Rhine-Main Neuroscience Network (rmn), Johannes Gutenberg University Hospital Mainz, Mainz, Germany; Department of Neurology, Research Center for Immunotherapy (FZI) and Focus Program Translational Neuroscience (FTN), Rhine Main Neuroscience Network (rmn^2^); Johannes Gutenberg University Hospital Mainz, Mainz, Germany; Department of Neurology, Charite – Universitatsmedizin Berlin, Berlin, Germany; Experimental and Clinical Research Center and NeuroCure Clinical Research Center, MaxDelbrueck Center for Molecular Medicine, Charite – Universitatsmedizin Berlin, Berlin, Germany; Division of Neuroimmunology, Department of Neurology, Heidelberg University, Heidelberg, Germany; Department of Neurology, TUM Klinikum Rechts der Isar, Technical University of Munich, Munich, Germany; Department of Neurology, University of Ulm, Ulm, Germany; Central Information Office German Competence Network of Multiple Sclerosis, CIO Marburg GmbH, Lohra, Germany; Neuroimmunological Section, Department of Neurology, University of Rostock, Rostock, Germany; Department of Neurology, Heinrich-Heine-Universität Düsseldorf Medizinische Fakultät, Dusseldorf, Nordrhein-Westfalen, Germany; Department of Neurology, Faculty of Medicine, University Hospital Augsburg, Augsburg, Germany; Department of Neurology, Hannover Medical School, Hanover, Germany; Molecular Neuroimmunology Group, Department of Neurology, University Hospital Heidelberg, Heidelberg, Germany; Department of Neurology, Leipzig University, Leipzig, Germany; Addiction Research and Clinical Epidemiology Unit, Centre for Health and Society, Medical Faculty and University Hospital Düsseldorf, Institute of General Practice, Heinrich Heine University Düsseldorf, Düsseldorf, Germany; Institut für Neuroimmunologie und Multiple Sklerose, University Medical Center Hamburg-Eppendorf, Hamburg, Germany

**Keywords:** alcohol consumption, disease progression, multiple sclerosis, prospective cohort study, smoking

## Abstract

**Background:**

Lifestyle factors, such as smoking cigarettes and alcohol consumption, can influence disease progression in Multiple Sclerosis (MS).

**Objective:**

This study aimed to investigate the associations of smoking cigarettes and alcohol consumption with clinical parameters in people with MS (pwMS).

**Design:**

This is a prospective cohort study, analysing data from the German NationMS cohort.

**Methods:**

We analysed data of 1374 patients recruited between 2010 and 2017. Main outcomes were Expanded Disability Status Scale (EDSS), Timed 25-Foot Walk (T25FW) and Paced Auditory Serial Addition Test (PASAT). Secondary outcomes were number of T2 and GD+ lesions. We conducted logistic and linear mixed model regression, adjusted for age, sex, educational level, MS subtype.

**Results:**

Smoking cigarettes was associated with increased odds of reaching pre-defined cut-off values for EDSS and T25FW, as well as with increased mean differences across follow-ups. Alcohol consumption was associated with decreased odds of reaching the cut-off for EDSS and T25FW. Findings for PASAT were less consistent. We found no significant associations for T2 and GD+ lesions.

**Conclusion:**

This study provides further evidence that smoking cigarettes accelerates MS progression and that pwMS should be advised to quit smoking. However, this negative impact could not be demonstrated for alcohol consumption. Reverse causation cannot be ruled out and further research in this area is warranted.

**Trial registration:**

Instead of registration in a primary trial registry, a comprehensive study protocol was published a priori and is available online: https://osf.io/3xscy.

## Plain language summary

How smoking and alcohol consumption affect the progression of multiple sclerosis

**Background:** Lifestyle habits, such as smoking and drinking alcohol, can influence how Multiple Sclerosis (MS) progresses over time. In this study, we wanted to investigate whether smoking and alcohol consumption are linked to changes in physical and mental health in people living with MS.

What did the researchers do?

We analyzed data from 1,374 patients who were part of a large German long-term study (the NationMS cohort) between 2010 and 2017. We focused on three main areas: the overall level of disability, walking speed, and mental sharpness (memory and concentration). We also looked at MRI brain scans to see if lifestyle factors were linked to the number of MS-related spots or scars (lesions) in the brain.

What were the main findings?

Our results showed that smoking was linked to a higher chance of worsening physical disability and slower walking speeds. Patients who smoked reached certain stages of disability sooner than non-smokers. Interestingly, alcohol consumption was associated with a lower risk of physical decline in this group. For mental sharpness, the results were not very clear. We found no significant link between smoking or alcohol and the number of lesions visible on MRI scans.

What do these findings mean?

This study provides more evidence that smoking can speed up the progression of MS. Therefore, people with MS should be encouraged to quit smoking to help manage their condition. While we did not find a negative impact from alcohol consumption, we cannot be certain that alcohol is ‘protective’. It is possible that people who feel healthier are simply more likely to drink alcohol, while those who feel worse stop drinking (a concept called ‘reverse causation’). More research is needed to fully understand these links.

## Background

Multiple Sclerosis (MS) is a chronic, inflammatory autoimmune disease that affects the central nervous system and is caused by both genetic and environmental factors.^1,2^ In Germany, around 280,000 people are affected by the disease (as of 2024).^3^ Research in recent years has shown that the development and progression of MS can be influenced by modifiable risk factors.^4^ Two particularly relevant factors are tobacco smoking and alcohol consumption.

As of August 2025, 34% of individuals aged 14 and older in Germany were active smokers.^5^ Furthermore, approximately 10 L of pure alcohol per capita are consumed annually in the German population, with an estimated 8 million adults engaging in high-risk alcohol consumption (defined as a daily average of at least 12 g for women and 24 g for men).^6^

Precise data on smoking prevalence or alcohol consumption among people with MS (pwMS) has been scarce and can vary based on the setting and sample. A previous study from 2022 using data from the German National MS cohort with 1066 patients found that 31.6% reported smoking at study entrance.^7^ Another study, examining a German clinical cohort of 399 pwMS, reported that 24.4% were current tobacco smokers.^8^ Regarding alcohol consumption, data from two independent case-control studies from Sweden show that among pwMS 60%–77% reported to consume alcohol.^9^ Additionally, an international study of 2469 pwMS^10^ reported that 61.5% indicated low alcohol consumption (less than 15 g weekly, including non-drinkers) and 37.7% consumed moderate amounts. Less than 1% were high consumers (>210–315 g weekly) or binge drinkers (>75 g/day on any one occasion).

Both active and passive smoking have been linked to an increased risk of developing MS.^11–13^ Moreover, studies have identified additional adverse effects of smoking on MS-related outcome measures, including higher relapse rates, increased mortality and worse overall disease outcome based on Expanded Disability Status Scale (EDSS).^11,14–16^ Furthermore, results from a German multicentre trial, involving 234 pwMS (19% (*n* = 45) current smokers), indicated that non-smokers had a lower burden of T2 lesions compared to smokers (RR = 0.76, *p* = 0.033).^17^ Additionally, smoking is associated with a higher risk of depression, anxiety disorders and overall lower quality of life in pwMS.^18,19^ Furthermore, MS medications tend to be less effective in smokers.^20^

A possible association of alcohol consumption, a lifestyle factor that often coincides with smoking,^21^ has also been studied. Some studies have reported a positive association between moderate alcohol consumption and both disease progression and quality of life in pwMS.^10,22^ However, a study from Belgium^22^ showed these effects only in people with relapsing-remitting MS (RRMS) and not in those with progressive MS.

Therefore, the primary aim of this study was to evaluate the long-term impact of cigarette smoking and alcohol consumption on disability progression in patients with early-stage MS. We sought to determine whether these health behaviours are risk factors for clinical outcome as well as neuroimaging markers over a multi-year period.

## Materials and methods

The reporting of this study adheres to ‘The Strengthening the Reporting of Observational Studies in Epidemiology (STROBE) Statement: guidelines for reporting observational studies’.^23^ Prior to the study, a protocol with a detailed description of our intended analyses was published (https://osf.io/3xscy).

### Ethics approval

The cohort study German NationMS was approved by the ethics committee of Ruhr-University Bochum (registration no. 3714-10) on 29 July 2010, and consecutively by all local committees of participating centres. A list of the participating centres and their respective local committees can be found in the Supplemental Material.

### Study design, setting and participants

We analysed data from the German NationMS cohort.^24^ This multicentre prospective longitudinal observational cohort study includes data from over 5000 visits of 1374 adult patients from 22 study centres across Germany, who are all part of the German Competence Network MS (KKNMS). Recruitment took place between 2010 and 2017 and participants were followed for up to 6 years. Participants were enrolled early in the course of the disease, diagnosed with either Clinically Isolated Syndrome (CIS) according to Barkhof criteria^25^ or with RRMS according to McDonald criteria 2005 or 2010, and followed over several years. Participants had to be 18 years or older, and naïve to treatment with disease-modifying drugs to be eligible. Exclusion criteria included, therefore, the previous use of disease-modifying therapy, primary progressive MS and other progressive forms of MS, concurrent progressive neurological diseases and conditions interfering with the assessment plan (e.g. general contraindication for magnetic resonance imaging (MRI)). For this study, we used baseline data as well as follow-up data from years 2, 4 and 6.

### Measures variables

#### Exposures

Tobacco use was measured at baseline using a standardised self-report questionnaire as part of the structured medical history taken by health care staff, by asking all participants about their tobacco consumption giving them the answering options (1) no; (2) only occasionally, not regularly; (3) up to 5 cigarettes daily; (4) up to 6–10 cigarettes daily; (5) up to 11–20 cigarettes daily and (6) more than 20 cigarettes daily. Past or former smoking was not assessed. For the purpose of this study, the use of the word ‘smoking’ always refers to smoking cigarettes. When asked about their alcohol consumption, the following answering options were given: (1) no; (2) sometimes and (3) regularly. In this context, ‘sometimes’ refers to occasional consumption without a fixed schedule or high frequency, while ‘regularly’ implies consistent consumption, typically on a weekly or daily basis. These broad categories were chosen to ensure sufficient group sizes for robust analyses and to maintain consistency with the format of the underlying data source, since specific units of alcohol per occasion were not recorded with this questionnaire.

#### Main outcomes

This study analyses the effect of smoking and alcohol consumption on three main outcomes, which are commonly used to measure disease progression in pwMS.

The EDSS is a scale to quantify disability in pwMS^26^ and primarily reflects motor function, especially in higher ranges. This ordinal scale ranges from 0 (normal function) to 10 (death due to MS).

The Timed 25-Foot Walk (T25FW)^27^ and the Paced Auditory Serial Addition Test (PASAT-3)^28^ are both components of the MS Functional Composite, a validated tool to assess disability in pwMS across multiple domains.^29^ The T25FW assesses walking speed by measuring the time in seconds it takes a participant to walk 25 feet as quickly and safely as possible.

The PASAT-3 evaluates cognitive processing speed, attention and working memory. Participants can reach a score from 0 to 60, with higher scores indicating better performance.^28^

The assessment of all outcomes was performed by trained personnel at each of the 22 participating centres, which all consented to the included parameters and implemented the scales of the mentioned outcomes. The variables were measured during regular clinical visits (follow-up periods), following a specific assessment plan.^30^

#### Secondary outcomes

For our analysis, we included two key MRI parameters as secondary outcomes: the total number of T2 hyperintense lesions and the presence of gadolinium-enhancing (GD+) lesions. For the MRI acquisition, all involved centres used standardised imaging protocols to acquire conventional MRI images. For that purpose, different 3T scanners with a 32-channel receive-only head coil were used. The overall process has been described in more detail before.^31^

### Statistical methods

All analyses were conducted using SPSS (version 29.0.2.0, IBM, New York, USA).

As is common for cohort studies, no formal a priori power analysis was performed, as the study aimed to include all eligible patients within the NationMS cohort during the recruitment period to maximise the representativeness of the findings.

For the baseline assessment, continuous variables were summarised with mean and standard deviation, categorical variables with absolute and relative frequencies.

We categorised smoking behaviour at baseline into the following categories: Non-smoker = No cigarettes smoked (answer option 1); Light smoker = Only occasionally, not regularly (answer option 2) or up to 5 cigarettes daily (answer option 3); Medium smoker = 6–20 cigarettes daily (answer options 4 and 5); Heavy smoker = more than 20 cigarettes daily (answer option 6).

Alcohol consumption was categorised into non-drinker (answer option 1), occasional drinker (answer option 2) and regular drinker (answer option 3).

#### Logistic regression

We used logistic regression modelling to investigate the impact of smoking and alcohol consumption on disease progression, whether the respective outcome cut-off was met at any point during the follow-up period. In order to do so, we dichotomised our main outcome variables using clinically relevant cut-off values. Specifically, EDSS ⩾3 was selected as it represents the transition to moderate disability and is widely used and considered a reasonable benchmark for the onset of mild-to-moderate, often irreversible disability in MS; T25FW ⩾6 s was used as a validated marker for clinically meaningful mobility impairment and is associated with occupational disability, job change due to MS or cane use and PASAT-3 ⩽35 was chosen to identify patients with relevant cognitive deficits. These cut-offs have been validated as clinically meaningful in other studies.^32–36^ Using these thresholds allows for a clearer interpretation of the impact of smoking and alcohol consumption on reaching key disability milestones within the given follow-up period.

In the logistic regression models, the independent variables were smoking behaviour, and alcohol consumption at baseline. The dependent variable was a binary indicator reflecting whether the clinical cut-off was reached at least once across the four measurement time points (baseline, follow-up data from years 2, 4 and 6). We built one model for each outcome: EDSS, T25FW, PASAT-3, T2 lesions and GD+ lesions. Analysis was adjusted for age, sex, educational level and MS type. As described in our study protocol (https://osf.io/3xscy), the selection of covariates was informed by Directed Acyclic Graphs to ensure a theoretically grounded model and to avoid overadjustment. An interaction of smoking and alcohol consumption was included if *p*_interaction_ < 0.15. Adjusted odds ratios (OR) with 95% confidence intervals (95% CI) and *p*-values are reported.

Only participants who had either (1) reached the clinical cut-off at least once (at baseline or during at least one of the follow-ups) or (2) had complete data across all four time points were included in the analysis. Participants who had never met the clinical cut-off and for whom data were missing at one or more time points were excluded.

For a sensitivity analysis, we added ever-use of immunotherapy as a covariate to our regression models. Since all participants were treatment-naïve at baseline, this variable represents the initiation of immunotherapy during the follow-up period.

#### Mixed models

Additionally, we conducted linear mixed-effects models to examine the association of smoking and alcohol consumption on our primary outcomes, taking into account the measurement at multiple time points. The dependent variable was the change from baseline in the respective score. For the EDSS and T25FW, a positive value indicated clinical worsening (higher disability/slower speed), whereas for the PASAT, a positive value indicated improvement (better cognitive function). The participants were included as a random effect. Smoking/alcohol consumption, the baseline value of the respective score, age, sex, educational level, MS type and the time point were treated as fixed effects. Similar to the regression models, we only included interaction terms between smoking/alcohol consumption and time point, if the respective *p*-value was <0.15.

The contrasts between the categories of smoking/alcohol consumption of the respective score (change from baseline) as well as adjusted means with 95% CI and *p*-values are reported.

#### Handling of missing data

We assessed the number of missing values for baseline and follow-ups. Additionally, we report if baseline characteristics from missing cases of smoking and alcohol consumption differed from those with complete data. For that, we differentiated between responders (available data for all time points); part-responders (available data for two or three time points) and non-responders (available data for only one time point), and performed χ^2^-tests.

In the mixed models, missing values were implicitly replaced by direct maximum likelihood imputation.

## Results

### Sample characteristics

The study comprised 1374 individuals, whose baseline characteristics are summarised in Table 1. In total, 70.1% (*n* = 963) were female. 599 (43.6%) had CIS and 774 (56.3%) RRMS, while we had missing data for 1 single case. The mean (SD) EDSS score was 1.43 (0.99) at baseline, and 1.58 (1.28) after 6 years, while the median EDSS was 1.5 both at baseline and after 6 years. The mean (SD) time in seconds for the T25FW was 4.39 (1.43) at baseline and 4.67 (1.47) after 6 years, while the median time in seconds increased from 4.2 at baseline to 4.35 after 6 years. The mean (SD) PASAT-3 score was 45.87 (11.02) at baseline and 51.56 (8.53) after 6 years, while the median increased from 48 to 54, respectively. At baseline, 66.1% (*n* = 908) indicated that they did not smoke, while 32% (*n* = 439) reported currently smoking to varying degrees. For 1.9% (*n* = 27), we had missing data. Additionally, 26.6% (*n* = 365) reported never drinking alcohol, 67.6% (*n* = 929) reported drinking alcohol sometimes, and 4.2% (*n* = 58) reported drinking alcohol regularly. For alcohol consumption, we had missing data for 1.6% (*n* = 22) of cases.

**Table 1.** Baseline characteristics.

Baseline (*n* = 1374)	*N*	%
Sex		
Male	411	29.9
Female	963	70.1
Missings	0	0
Age		
18–29	559	40.7
30–45	613	44.6
46–59	184	13.4
60 and older	6	0.4
Missings	12	0.9
Education		
No degree	14	1.0
Lower secondary school	166	12.1
Intermediate secondary school	371	27.0
Upper secondary school	21	1.5
College entrance qualification	194	14.1
Higher education (abitur)	593	43.2
Missings	15	1.1
Tobacco use		
Non-smoker		
No	908	66.1
Light smoker		
Only occasionally, not regularly	98	7.1
Up to 5 cigarettes daily	47	3.4
Medium smoker		
6–10 cigarettes daily	118	8.6
11–20 cigarettes daily	134	9.8
Heavy smoker		
>20 cigarettes daily	42	3.1
Missings	27	1.9
Alcohol use		
No	365	26.6
Sometimes	929	67.6
Regularly	58	4.2
Missings	22	1.6
MS type		
CIS	599	43.6
RRMS	774	56.3
Missings	1	0.1
T2-lesions		
1–8	424	30.9
9 or more	941	68.5
Missings	9	0.7
GD+ lesions		
No	822	59.8
Yes	490	35.7
Missings	62	4.5
Immunotherapy (ever)		
No	307	22.3
Yes	1040	75.7
Missing	27	2.0
	Mean (SD)	Median
EDSS (*n* = 1373)	1.43 (0.99)	1.5
T25FW (*n* = 1356)	4.39 (1.43)	4.2
PASAT-3 (*n* = 1269)	45.87 (11.02)	48.0

CIS, clinically isolated syndrome; EDSS, Expanded Disability Status Scale; GD+, gadolinium-positive; MS, multiple sclerosis; PASAT, Paced Auditory Serial Addition Test; RRMS, relapsing remitting multiple sclerosis; SD, standard deviation; T25FW, timed 25-foot walk.

### Smoking

Table 2 shows the results from our regression models for each of our main and secondary outcomes. Every smoking group (light, medium, heavy) showed increased odds for ever reaching the EDSS cut-off of 3 within 6 years after baseline compared to non-smokers (OR_light_ = 1.80 (CI = 1.03; 3.13; *p* = 0.041); OR_medium_ = 1.29 (CI = 0.80; 2.07; *p* = 0.294); OR_heavy_ = 2.15 (CI = 0.77; 6.05; *p* = 0.146)). Similarly, odds for reaching the T25FW cut-off of 6 were increased for all smoking groups compared to non-smokers (OR_light_ = 1.85 (CI = 1.01; 3.41; *p* = 0.047); OR_medium_ = 1.02 (CI = 0.60; 1.75; *p* = 0.930); OR_heavy_ = 1.54 (CI = 0.52; 4.54; *p* = 0.432)). Findings for the PASAT were less consistent, with the odds for reaching the cut-off of 35 being increased for light and heavy smokers, but decreased for medium smokers compared to non-smokers (OR_light_ = 1.64 (CI = 0.74; 3.64; *p* = 0.224); OR_medium_ = 0.88 (CI = 0.48; 1.61; *p* = 0.678); OR_heavy_ = 1.31 (CI = 0.48; 4.75; *p* = 0.678)). We could not identify an association of the number of new T2 or GD+ lesions with the smoking status. Results did not differ significantly when additionally adjusting the regression models with the variable ever-use of immunotherapy (Supplemental Material).

**Table 2.** Results from the logistic regression models for each outcome, indicating the odds of reaching the pre-defined cut-off value within the 6-year follow-up period.

Group	EDSS			T25FW			PASAT-3			T2 lesions			GD+ lesions		
*N*	654			595			393			1104			599		
Groups	OR	CI	Sig.	OR	CI	Sig.	OR	CI	Sig.	OR	CI	Sig.	OR	CI	Sig.
Non-smoker (ref)															
Light smoker	1.80	1.03; 3.13	0.041	1.85	1.01; 3.41	0.047	1.64	0.74; 3.64	0.224	1.55	0.54; 4.47	0.422	0.88	0.37; 2.13	0.780
Medium smoker	1.29	0.80; 2.07	0.294	1.02	0.60; 1.75	0.930	0.88	0.48; 1.61	0.678	1.38	0.58; 3.27	0.466	0.54	0.26; 1.09	0.086
Heavy smoker	2.15	0.77; 6.05	0.146	1.54	0.52; 4.54	0.432	1.31	0.36; 4.75	0.678	/	/	0.998	0.75	0.20; 2.84	0.667
Non-drinker (ref)															
Occasional drinkers	0.49	0.33; 0.74	0.001	0.54	0.35; 0.85	0.007	0.47	0.28; 0.81	0.005	0.84	0.43; 1.67	0.625	0.68	0.33; 1.41	0.296
Regular drinkers	0.53	0.21; 1.33	0.178	0.11	0.01; 0.91	0.040	0.57	0.16; 2.06	0.392	0.99	0.21; 4.81	0.996	0.46	0.11; 1.97	0.295

Adjusted for: drinking behaviour/smoking behaviour, age, sex, education, MS type.

CI, 95% confidence interval; EDSS, Expanded Disability Status Scale; GD, gadolinium; OR, odds ratio; PASAT-3, Paced Auditory Serial Addition Test; ref. reference group; Sig., significance; T25FW, timed 25-foot walk.

**Figure 1.**
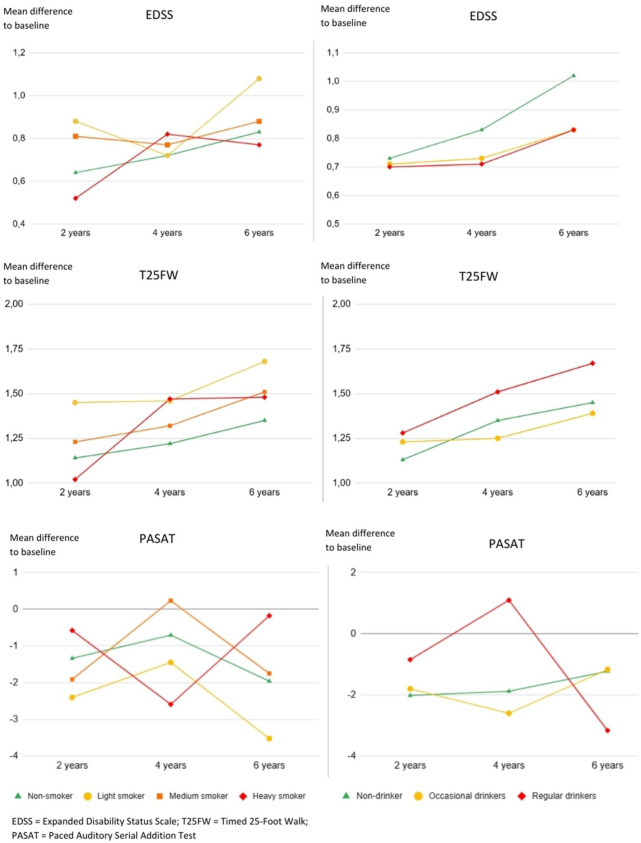
EMMs for each main outcome. EMMs, estimated marginal means.

Table 3 shows the results from the linear mixed models, looking at smoking behaviour and the mean difference of the respective score compared to baseline. Figure 1 shows estimated marginal means for each main outcome. For EDSS, the mean difference for light smokers compared to non-smokers was 0.25 (CI = 0.05; 0.45; *p* = 0.02) at 2-year follow-up (*t*_1_), −0.01 (CI = −0.22; 0.21; *p* = 0.95) at 4-year follow-up (*t*_2_) and 0.24 (CI = 0.01; 0.47; *p* = 0.04) at 6-year follow-up (*t*_3_). We also found increased mean differences for medium smokers compared to non-smokers across all time-points (*t*_1_ = 0.17 (CI = 0.01; 0.33; *p* = 0.04); *t*_2_ = 0.04 (CI = −0.13; 0.22; *p* = 0.62); *t*_3_ = 0.04 (CI = −0.14; 0.23; *p* = 0.65). For heavy smokers, the mean difference in EDSS at *t*_2_ compared to baseline was negative (−0.11 (CI = −0.50; 0.28; *p* = 0.57)) but all coefficients for this group comparison were non-significant. The interaction terms between time and both PASAT and T25FW were excluded from the models (*p* > 0.15). Hence, the reported values refer to the mean difference of the respective outcome across all follow-up time points compared to baseline. For PASAT, light and medium smokers showed negative mean differences compared to non-smokers (light = −1.10 (CI = −2.95; 0.75; *p* = 0.24); medium = −0.03 (−1.53; 1.46; *p* = 0.97)), while heavy smokers showed a positive mean difference (0.10 (CI = −3.66; 3.86; *p* = 0.96)). However, all coefficients were non-significant. For the T25FW, all smoking groups had a positive mean difference compared to baseline (light = 0.30 (CI = 0.01; 0.60; *p* = 0.05); medium = 0.11 (CI = −0.14; 0.35; *p* = 0.40); heavy = 0.05 (CI = −0.54; 0.64; *p* = 0.88)).

**Table 3.** Mixed model results for drinking and smoking behaviour, indicating the mean difference of the respective outcome score compared to baseline.

Groups		EDSS			T25FW			PASAT		
Fixed effects		Mean difference	*p*-Value	95% CI	Mean difference	*p*-Value	95% CI	Mean difference	*p*-Value	95% CI
Smoking behaviour										
Light vs non-smoker					0.30	0.05	0.01; 0.60	−1.10	0.24	−2.95; 0.75
Medium vs non-smoker					0.11	0.40	−0.14; 0.35	−0.03	0.97	−1.53; 1.46
Heavy vs non-smoker					0.05	0.88	−0.54; 0.64	0.10	0.96	−3.66; 3.86
Interaction with time	*t*	*p* = 0.007			*p* = 0.934			*p* = 0.336		
Light vs non-smoker	2	0.25	0.02	0.05; 0.45						
	3	−0.01	0.95	−0.22; 0.21						
	4	0.24	0.04	0.01; 0.47						
Medium vs non-smoker	2	0.17	0.04	0.01; 0.33						
	3	0.04	0.62	−0.13; 0.22						
	4	0.04	0.65	−0.14; 0.23						
Heavy vs non-smoker	2	−0.11	0.57	−0.50; 0.28						
	3	0.10	0.62	−0.31; 0.51						
	4	0.07	0.77	−0.55; 0.41						
Drinking behaviour										
Occasional drinker vs non-drinker		−0.08	0.20	−0.21; 0.04	0.01	0.97	−0.21; 0.22			
Regular drinker vs non-drinker		−0.09	0.52	−0.38; 0.20	0.16	0.51	−0.32; 0.65			
Interaction with time	*t*	*p* = 0.364			*p* = 0.511			*p* = 0.064		
Occasional drinker vs non-drinker	2							0.19	0.79	−1.21; 1.59
	3							−0.68	0.42	−2.33; 0.96
	4							0.08	0.93	−1.63; 1.79
Regular drinker vs non-drinker	2							1.10	0.50	−2.10; 4.30
	3							3.01	0.12	−0.77; 6.80
	4							−1.85	0.41	−6.20; 2.51

Adjusted for: smoking behaviour/drinking behaviour, age, sex, education, MS type.

BL, baseline; CI, 95% confidence interval; EDSS, Expanded Disability Status Scale; MS, multiple sclerosis; PASAT-3, Paced Auditory Serial Addition; *t*, timepoint (2 = 2 years after baseline; 3 = 4 years after baseline; 4 = 6 years after baseline); T25FW, timed 25 foot walk.

### Alcohol consumption

For alcohol consumption, ORs were decreased for both occasional and regular drinkers compared to non-drinkers within 6 years for ever reaching the EDSS cut-off (OR_occasional_ = 0.49 (CI = 0.33; 0.74; *p* = 0.001); OR_regular_ = 0.54 (CI = 0.21; 1.33; *p* = 0.178)), the T25FW cut-off (OR_occasional_ = 0.54 (CI = 0.35; 0.85; *p* = 0.007); OR_regular_ = 0.11 (CI = 0.01; 0.91; *p* = 0.040)) and the PASAT cut-off (OR_occasional_ = 0.47 (CI = 0.28; 0.81; *p* = 0.005); OR_regular_ = 0.57 (CI = 0.16; 2.06; *p* = 0.392)). For T2 and GD+ lesions, trends were similar, but all ORs were non-significant (see Table 2). As for smoking, results did not differ significantly when additionally adjusting the regression models with the variable ever-use of immunotherapy (results not shown).

For EDSS, mean differences across all time points for both occasional and regular drinkers compared to non-drinkers were negative (occasional = −0.08 (CI = −0.21; 0.04; *p* = 0.20); regular = −0.09 (CI = −0.38; 0.20; *p* = 0.52)). On the other hand, mean differences were positive for the T25FW (occasional = 0.01 (CI = −0.21; 0.22; *p* = 0.97); regular = 0.16 (CI = −0.32; 0.65; *p* = 0.51)). For the PASAT we included the interaction term. However, none of the interactions between occasional and regular drinkers, and each time point compared to baseline reached significance, with mean differences indicating no clear tendency in one or the other direction (see Table 3).

### Missings

Table 4 shows the results from the non-responder analysis, where we compared responders, non-responders and part-responders for the variables of smoking and alcohol consumption in regard to our baseline characteristics to check for possible differences between groups using χ^2^ tests. For both smoking and alcohol consumption we found differences in age groups (*p*_smoking_ = 0.011; *p*_drinking_ = 0.001): In both groups (smoking and alcohol consumption), part- and non-responders were more likely to be 18–29 years old and less likely to be 30–45 or 46–59 years old compared to responders. Differences within groups for all other variables were less pronounced.

**Table 4.** Results from the non-responder analysis using χ^2^-tests; numbers in %.

Variable	Smoking				Alcohol			
	*p*-Value	Responder	Part-responder	Non-responder	*p*-Value	Responder	Part-responder	Non-responder
Sex (*n* = 1367)	0.175				0.209			
Male		32.1	29.5	26.1		32.1	29.3	26.4
Female		67.9	70.5	73.9		67.9	70.7	73.6
Age (*n* = 1355)	0.011				0.001			
18–29		37.2	42.3	46.3		35.7	43.7	46.6
30–45		46.3	44.5	42.8		47.1	43.7	42.7
46–59		16.3	12.2	10.9		17.0	11.6	10.7
60 and older		0.2	1.0	0.0		0.2	1.0	0.0
Education (*n* = 1353)	0.654				0.558			
No degree		0.7	0.8	1.9		0.7	0.8	1.9
Lower secondary school		11.6	13.6	10.8		11.6	13.9	10.3
Intermediate secondary school		26.5	26.8	29.9		26.8	26.3	30.2
Upper secondary school		1.4	1.9	1.3		1.6	1.4	1.6
College entrance qualification		13.5	14.4	15.0		13.2	14.9	15.1
Higher education (Abitur)		46.2	42.5	41.1		46.0	42.7	40.8
MS type (*n* = 1366)	0.491				0.516			
CIS		42.0	45.6	43.1		42.2	45.6	42.7
RRMS		58.0	54.4	56.9		57.8	54.4	57.3
T2-Lesions (*n* = 1358)	0.286				0.726			
1–8		32.5	28.3	32.3		31.6	29.7	32.1
9 or more	0.969	67.5	71.7	67.7		68.4	70.3	67.9
GD+ lesions (*n* = 1307)					0.996			
No		62.4	62.6	63.2		62.5	62.7	62.9
Yes		37.6	37.4	36.8		37.5	37.3	37.1
EDSS (*n* = 1366)	0.108				0.068			
Mean (SD)		1.45 (1.00)	1.36 (0.99)	1.50 (0.96)		1.46 (1.01)	1.35 (0.98)	1.51 (0.97)
T25FW (*n* = 1349)	0.195				0.156			
Mean (SD)		4.41 (1.67)	4.30 (0.97)	4.49 (1.53)		4.41 (1.67)	4.30 (0.97)	4.50 (1.56)
PASAT-3 (*n* = 1263)	0.292				0.195			
Mean (SD)		46.45 (10.68)	45.38 (11.32)	45.63 (11.19)		46.52 (10.64)	45.29 (11.31)	45.58 (11.24)

CIS, clinically isolated syndrome; EDSS, Expanded Disability Status Scale; GD+, gadolinium-positive; MS, multiple sclerosis; PASAT, paced auditory serial addition test; RRMS, relapsing remitting multiple sclerosis; SD, standard deviation; T25FW, timed 25-foot walk.

## Discussion

Our analysis in a large nation-wide observational cohort of initially treatment-naïve pwMS shows that smokers had increased odds of reaching the pre-defined cut-off values for EDSS and T25FW, as well as higher mean differences (i.e. faster functional deterioration) across follow-ups compared to non-smokers. Conversely, alcohol consumption appeared inversely associated with these same clinical endpoints. Our findings for PASAT were less consistent for both groups, and critically, neither lifestyle factor demonstrated significant associations with T2 lesion burden or active GD+ lesions on MRI. Results did not differ meaningfully when regression models were adjusted for ever-use of immunotherapy.

The findings regarding smoking are consistent with the existing literature claiming an impact of smoking on MS progression and disability.^11^ While some of our ORs were non-significant, we believe that these results are highly informative and shouldn’t be ignored,^37,38^ since the ORs for different smoking groups often point in the same direction, suggestion a consistent trend, despite the lack of significance in some cases . For example, some studies also found an increased risk for pwMS who smoke of reaching an EDSS score of 4 and 6 earlier compared to pwMS who do not smoke,^39^ or slower walking speeds in pwMS who ever smoked compared to those who have not.^40^ Therefore, our findings in combination with existing evidence suggesting that smoking cessation is beneficial for the disease course of pwMS,^39^ further support the recommendation that pwMS should be advised to stop smoking during all stages of their disease. However, our study found no clear evidence that smoking affects the evolution of T2 or GD lesions. The literature here reports mixed results, with some studies reporting increased lesion burden in people who smoke compared to people who do not,^11^ while other studies did not identify a clear relationship.^41,42^ While it is known that components of cigarette smoke, like hydrogen cyanide or carbon monoxide, promote neurodegeneration by damaging the myelin sheath or entire axons,^43^ the lack of associations for T2 and GD lesions might suggest that smoking-induced damage is mediated more through other neurodegenerative processes rather than acute inflammatory activity. Furthermore, the categorical assessment of smoking intensity in our study might lack the sensitivity to detect a dose-dependent relationship with MRI activity, especially if the pro-inflammatory effects of smoking are more subtle in the short term. For now, based on our results and the literature, it cannot be stated that smoking is associated with an increase in lesion formation.

In contrast, our study found, in some instances, a seemingly protective association between alcohol consumption and MS outcomes, a finding also supported by some other studies.^9,10,22^ Nevertheless, evidence for the effect of alcohol is less clear than for smoking. Here, it is crucial to consider the possibility of reverse causation, as healthier individuals might be more likely to consume alcohol compared to individuals who are more affected by their disease.^44^ This might happen when individuals are becoming more aware of their disease burden as it progresses, and are consequently reducing the number or frequency of behaviours that are perceived as unhealthy. But, on the contrary, it could be argued that pwMS with higher disease burden might start drinking more due to sorrow or distress over their illness. Furthermore, the potential role of residual confounding by socioeconomic status should be considered, as a higher alcohol consumption is often associated with a higher socioeconomic status,^45,46^ which, in turn, is linked to better disability outcomes in MS.^47^ It should also be mentioned that alcohol has an immunomodulatory and sometimes anti-inflammatory effect, but its impact on MS is inconsistent and strongly dose-dependent.^48^ Hence, current evidence does not support using alcoholic beverages as a deliberate anti-inflammatory ‘therapy’ in MS, and potential benefits must be weighed against substantial known risks.^48^

### Limitations and strengths

This study has some limitations. The size of subgroups, such as heavy smokers and regular drinkers, was small, and the number of participants with complete data decreased for each follow-up, which may have limited our ability to detect more reliable effects in these populations and might partially explain why, for instance, some ORs of medium smokers were lower compared to light smokers in our logistic regression models (Table 2). For this reason, we also did not consider changes in smoking and alcohol consumption status over the study period, since this would have further reduced potential sub-group size and statistical robustness. Also, we acknowledge that the categorisation of smoking and alcohol consumption is rather broad and that a more detailed categorisation would have allowed for more specific and nuanced analysis of dose-response relations. Yet, a more detailed categorisation would have also resulted in even smaller subgroups, which would have been unfit for the conduction of meaningful analysis. A similar limitation underlies our MRI analysis, where available data would allow only the differentiation between ‘1–8 T2 lesions’ or ‘9 or more T2 lesions’, or ‘yes’ or ‘no’ for the presence of GD+ lesions. While these broad classifications may introduce a degree of misclassification bias, they were necessary to maintain consistent reporting across the 6-year follow-up period. Furthermore, our cohort consists exclusively of individuals with CIS and RRMS, who were enrolled early in their disease course, and most of them had not reached major disability milestones during the follow-up. Therefore, our results cannot be generalised to all pwMS, especially not those with progressive forms of MS. Also, by choosing to alternate outcome measures in contrast to our fixed cut-off values for logistic regression and utilising alternative methods for analysis, future research could further refine the understanding of lifestyle-induced changes in MS trajectory. For example, a survival analysis would allow for an evaluation of the ‘time-to-event’ and account for the temporal dynamics of progression, which would provide additional insights into how factors like smoking and alcohol use influence the velocity of disability accumulation. Additionally, our non-responder analysis indicated a higher attrition rate among younger participants (aged 18–29). This could have introduced selection bias, since younger patients might have a shorter disease duration and less additional progression within the follow-up period. Despite having access to longitudinal data, a 6-year follow-up is not sufficient to fully capture long-term effects of smoking and alcohol on disease progression.

One considerable strength of our real-world observation study is its large overall sample size with access to data from several follow-up visits. The multicentre nature of this national cohort strengthens its external validity and helps mitigate potential biases. The use of logistic regression as well as linear mixed models allowed for a detailed and specific look on the effects of alcohol and smoking on MS over time.

## Conclusion

Our findings largely corroborate existing evidence that smoking accelerates MS disease progression, reinforcing the clinical imperative to strongly advise all pwMS to cease smoking. The role of alcohol remains less certain and a more cautious interpretation of the possibly positive effects is warranted due to the possibility of reverse causality. Further prospective research is required to clarify the complex relationship between alcohol and MS.

## Supplemental Material

sj-docx-1-tan-10.1177_17562864261464304 – Supplemental material for Impact of tobacco and alcohol consumption on disease progression and MRI in people with multiple sclerosis: results of the prospective cohort study NationMS Supplemental material, sj-docx-1-tan-10.1177_17562864261464304 for Impact of tobacco and alcohol consumption on disease progression and MRI in people with multiple sclerosis: results of the prospective cohort study NationMS by Alex Maximilian Keller, Annika Möhl, Anke Salmen, Ralf Gold, Pia Renk, Luisa Klotz, Stefan Bittner, Vinzenz Fleischer, Friedemann Paul, Klemens Ruprecht, Katrin Giglhuber, Hayrettin Tumani, Gisela Antony, Uwe Zettl, Sven G. Meuth, Antonios Bayas, Corinna Trebst, Brigitte Wildemann, Florian Then Bergh, Daniel Kotz and Christoph Heesen in Therapeutic Advances in Neurological Disorders

sj-docx-2-tan-10.1177_17562864261464304 – Supplemental material for Impact of tobacco and alcohol consumption on disease progression and MRI in people with multiple sclerosis: results of the prospective cohort study NationMS Supplemental material, sj-docx-2-tan-10.1177_17562864261464304 for Impact of tobacco and alcohol consumption on disease progression and MRI in people with multiple sclerosis: results of the prospective cohort study NationMS by Alex Maximilian Keller, Annika Möhl, Anke Salmen, Ralf Gold, Pia Renk, Luisa Klotz, Stefan Bittner, Vinzenz Fleischer, Friedemann Paul, Klemens Ruprecht, Katrin Giglhuber, Hayrettin Tumani, Gisela Antony, Uwe Zettl, Sven G. Meuth, Antonios Bayas, Corinna Trebst, Brigitte Wildemann, Florian Then Bergh, Daniel Kotz and Christoph Heesen in Therapeutic Advances in Neurological Disorders

sj-docx-3-tan-10.1177_17562864261464304 – Supplemental material for Impact of tobacco and alcohol consumption on disease progression and MRI in people with multiple sclerosis: results of the prospective cohort study NationMS Supplemental material, sj-docx-3-tan-10.1177_17562864261464304 for Impact of tobacco and alcohol consumption on disease progression and MRI in people with multiple sclerosis: results of the prospective cohort study NationMS by Alex Maximilian Keller, Annika Möhl, Anke Salmen, Ralf Gold, Pia Renk, Luisa Klotz, Stefan Bittner, Vinzenz Fleischer, Friedemann Paul, Klemens Ruprecht, Katrin Giglhuber, Hayrettin Tumani, Gisela Antony, Uwe Zettl, Sven G. Meuth, Antonios Bayas, Corinna Trebst, Brigitte Wildemann, Florian Then Bergh, Daniel Kotz and Christoph Heesen in Therapeutic Advances in Neurological Disorders

## Declarations

**Ethics approval and consent to participate:** The cohort study German NationMS was approved by the ethics committee of Ruhr-University Bochum (registration no. 3714-10), and consecutively by all local committees of participating centres. All study participants provided written informed consent.

**Consent for publication:** Not applicable.

**Availability of data and materials:** Data are available upon reasonable request.
