# Supplementary material for: Impact of tobacco and alcohol consumption on disease progression and MRI in people with multiple sclerosis: results of the prospective cohort study NationMS
Source: Ther Adv Neurol Disord. 2026 Jul 29;19:17562864261464304. doi: 10.1177/17562864261464304 (PMC13424504; doi:10.1177/17562864261464304)
Supplement: sj-docx-2-tan-10.1177_17562864261464304 – Supplemental material for Impact of tobacco and alcohol consumption on disease progression and MRI in people with multiple sclerosis: results of the prospective cohort study NationMS [file sj-docx-2-tan-10.1177_17562864261464304.docx]

**Supplement 1: Sensitivity Analysis for the Regression Models including Disease Modifying Therapy as an Adjusting Variable**

Supplement Table 1: Results from the logistic regression models for each outcome, indicating the odds of reaching the predefined cut-off value within the six-year follow-up period, including Disease Modifying Therapy (DMT) as an adjusting variable

|  | **EDSS** | | | **T25FW** | | | **PASAT-3** | | | **T2 lesions** | | | **GD+ lesions** | | |
| --- | --- | --- | --- | --- | --- | --- | --- | --- | --- | --- | --- | --- | --- | --- | --- |
| N | 654 | | | 595 | | | 393 | | | 1104 | | | 599 | | |
| **Groups** | **OR** | **CI** | **Sig.** | **OR** | **CI** | **Sig.** | **OR** | **CI** | **Sig.** | **OR** | **CI** | **Sig.** | **OR** | **CI** | **Sig.** |
| Non-smoker (ref) |  |  |  |  |  |  |  |  |  |  |  |  |  |  |  |
| Light smoker | 1.80 | 1.03; 3.16 | .039 | 1.86 | 1.01; 3.42 | .046 | 1.77 | 0.80; 3.95 | .160 | 1.55 | 0.52; 4.37 | .446 | 0.89 | 0.37; 2.18 | .802 |
| Medium smoker | 1.29 | 0.80; 2.09 | .280 | 1.02 | 0.60; 1.75 | .931 | 0.88 | 0.47; 1.62 | .671 | 1.39 | 0.59; 3.30 | .454 | 0.54 | 0.26; 1.10 | .539 |
| Heavy smoker | 1.90 | 0.71; 5.76 | .189 | 1.56 | 0.53; 4.63 | .424 | 0.95 | 0.25; 3.55 | .934 | / | / | .998 | 0.58 | 0.15; 2.26 | .579 |
| Non-drinker (ref) |  |  |  |  |  |  |  |  |  |  |  |  |  |  |  |
| Occasional drinkers | 0.50 | 0.34; 0.76 | .001 | 0.54 | 0.35; 0.85 | .007 | 0.49 | 0.29; 0.83 | .008 | 0.86 | 0.43; 1.70 | .662 | 0.74 | 0.35; 1.53 | .411 |
| Regular drinkers | 0.55 | 0.22; 1.36 | .194 | 0.11 | 0.01; 0.90 | .040 | 0.60 | 0.16; 2.25 | .447 | 1.00 | 0.21; 4.83 | .996 | 0.40 | 0.09; 1.79 | .231 |
| Adjusted for: drinking behaviour / smoking behaviour, age, sex, education, MS type, DMT (disease modifying therapy) OR = Odds Ratio; ref = reference group; Sig. = Significance; CI = 95% Confidence Interval; EDSS = Expanded Disability Status Scale; T25FW = Timed 25-Foot Walk; PASAT-3 = Paced Auditory Serial Addition Test; GD = Gadolinium | | | | | | | | | | | | | | | |

Table 2: Results from the logistic regression models for each outcome, indicating the odds of reaching the predefined cut-off value within the six-year follow-up period

|  | **EDSS** | | | **T25FW** | | | **PASAT-3** | | | **T2 lesions** | | | **GD+ lesions** | | |
| --- | --- | --- | --- | --- | --- | --- | --- | --- | --- | --- | --- | --- | --- | --- | --- |
| N | 654 | | | 595 | | | 393 | | | 1104 | | | 599 | | |
| **Groups** | **OR** | **CI** | **Sig.** | **OR** | **CI** | **Sig.** | **OR** | **CI** | **Sig.** | **OR** | **CI** | **Sig.** | **OR** | **CI** | **Sig.** |
| Non-smoker (ref) |  |  |  |  |  |  |  |  |  |  |  |  |  |  |  |
| Light smoker | 1.80 | 1.03; 3.13 | .041 | 1.85 | 1.01; 3.41 | .047 | 1.64 | 0.74; 3.64 | .224 | 1.55 | 0.54; 4.47 | .422 | 0.88 | 0.37; 2.13 | .780 |
| Medium smoker | 1.29 | 0.80; 2.07 | .294 | 1.02 | 0.60; 1.75 | .930 | 0.88 | 0.48; 1.61 | .678 | 1.38 | 0.58; 3.27 | .466 | 0.54 | 0.26; 1.09 | .086 |
| Heavy smoker | 2.15 | 0.77; 6.05 | .146 | 1.54 | 0.52; 4.54 | .432 | 1.31 | 0.36; 4.75 | .678 | / | / | .998 | 0.75 | 0.20; 2.84 | .667 |
| Non-drinker (ref) |  |  |  |  |  |  |  |  |  |  |  |  |  |  |  |
| Occasional drinkers | 0.49 | 0.33; 0.74 | .001 | 0.54 | 0.35; 0.85 | .007 | 0.47 | 0.28; 0.81 | .005 | 0.84 | 0.43; 1.67 | .625 | 0.68 | 0.33; 1.41 | .296 |
| Regular drinkers | 0.53 | 0.21; 1.33 | .178 | 0.11 | 0.01; 0.91 | .040 | 0.57 | 0.16; 2.06 | .392 | 0.99 | 0.21; 4.81 | .996 | 0.46 | 0.11; 1.97 | .295 |
| Adjusted for: drinking behaviour / smoking behaviour, age, sex, education, MS type OR = Odds Ratio; ref = reference group; Sig. = Significance; CI = 95% Confidence Interval; EDSS = Expanded Disability Status Scale; T25FW = Timed 25-Foot Walk; PASAT-3 = Paced Auditory Serial Addition Test; GD = Gadolinium | | | | | | | | | | | | | | | |
