# Supplementary material for: Impact of tobacco and alcohol consumption on disease progression and MRI in people with multiple sclerosis: results of the prospective cohort study NationMS
Source: Ther Adv Neurol Disord. 2026 Jul 29;19:17562864261464304. doi: 10.1177/17562864261464304 (PMC13424504; doi:10.1177/17562864261464304)
Supplement: sj-docx-3-tan-10.1177_17562864261464304 – Supplemental material for Impact of tobacco and alcohol consumption on disease progression and MRI in people with multiple sclerosis: results of the prospective cohort study NationMS [file sj-docx-3-tan-10.1177_17562864261464304.docx]

**NationMS cohort study – Ethic approvals**

| **City** | **Name** | **Number** | **Date (month/year)** |
| --- | --- | --- | --- |
| Augsburg | Ethics Committee of the Ludwig Maximilian University of Munich | 7/11113 | XX/2010 |
| Bad Mergentheim | Ethics Committee of the Baden-Württemberg State Medical Association | B-F-2010-051 | 12/2010 |
| Berlin (Charite) | Ethics Committee of the Charité – Universitätsmedizin Berlin | The ethics committee of the Charité generally waives the requirement for secondary follow-up approval if an ethical approval from another university is already in place. | n/a |
| Bochum | Ethics Committee of the Faculty of Medicine, Ruhr University Bochum | 3714-10 | 07/2010 |
| Düsseldorf | Ethics Committee of the Faculty of Medicine, Heinrich Heine University Düsseldorf | 3464 | 12/2010 |
| Erfurt | Ethics Committee of the Thuringia State Medical Association | 30383/2010/128 | 12/2010 |
| Erlangen | Ethics Committee of the Friedrich Alexander University Erlangen Nuremberg | 4589-CH | XX/2010 |
| Hamburg | Ethics Committee of the Hamburg Medical Association | MC-265/10 | XX/2010 |
| Hannover | Ethics Committee of the Hannover Medical School | 820-2010 | XX/2010 |
| Heidelberg | Ethics Committee of the Faculty of Medicine, University of Heidelberg | S-370/2010 | XX/2010 |
| Leipzig | Ethics Committee of the Faculty of Medicine University of Leipzig | 037-11-24012011 | 02/2011 |
| LMU Munich | Ethics Committee of the Faculty of Medicine, Ludwig Maximilian University of Munich | 363-10 | 01/2011 |
| Mainz | Ethics Committee of the Rhineland-Palatinate State Medical Association | 837.363.10 | 06/2011 |
| Marburg | Ethics Committee of the Faculty of Medicine, Philipps University of Marburg | 174/10 | 07/2010 |
| MPI Munich | Ethics Committee of the Faculty of Medicine, Ludwig Maximilian University of Munich | 326/10 | 08/2011 |
| Munster | Ethics Committee of the Westphalia-Lippe Medical Association and the University of Münster | 2010-378-b-S | XX/2010 |
| Rostock | Ethics Committee of the Faculty of Medicine, University of Rostock | A 2010-0095 | 11/2010 |
| TU Munich | Ethics Committee of the Faculty of Medicine, Technical University of Munich | 2899/10 | 08/2010 |
| Tübingen | Ethics Committee of the Medical Faculty of the University of Tübingen | 127/2011/BO2 | XX/2011 |
| Ulm | Ethics Committee of the University of Ulm | 276/10 | 11/2010 |
